# “*Padon pa geri maleng*” (“Sorry doesn't heal the scars”)

**DOI:** 10.4269/ajtmh.16-0859

**Published:** 2017-01-11

**Authors:** Claire Panosian Dunavan

**Affiliations:** 1 University of California, Los Angeles, California. E-mail: cpanosian@mednet.ucla.edu

Disasters, discord, poverty. What hardened heart doesn't ache for Haiti? Fifty years after Columbus's arrival, Hispaniola's natives were decimated by disease; by the mid-1700s, French overlords were exploiting Haiti's African slaves. Then came revolution, independence, and more upheaval. Finally, in 2004, after its first democratically elected president was ousted by a coup, the United Nations sent an international force to Haiti.

Six years later came the double whammy from hell. In January 2010, an earthquake killed or injured half a million Haitians; in October 2010, a massive fecal spill sowed *Vibrio cholerae* in the Artibonite River.

Today, no one denies that Haiti's modern epidemic stemmed from that tragic leak of sewage from a UN peacekeepers' camp. But it was not until August 2016 that UN Secretary General Ban Ki-moon admitted the fact. When he did, retired University of California, Los Angeles epidemiologist Ralph Frerichs was ready. Having recently published *Deadly River—Cholera and Cover-up in Post-Earthquake Haiti*, Frerichs wrote in the *Boston Globe*: “It is not enough that the United Nations is finally beginning to acknowledge its involvement in the lethal cholera epidemic in Haiti. Now it must urgently do everything in its power to eliminate cholera in Haiti before thousands more die.”

(The comments were sadly prescient. In the wake of Hurricane Matthew, cholera's grisly toll of withered, gray corpses can only continue to rise.)

In *Deadly River*, Frerichs largely channels Renaud Piarroux, a French infectious diseases doctor who previously battled cholera in Comoros and the former Zaire. Days after cholera's Caribbean touchdown, Piarroux flew from Marseille to Port-au-Prince at the request of the Haitian government. And here the story begins.

In addition to Piarroux's personal investigations and uncensored thoughts, what distinguishes *Deadly River* is its deep dissection of cholera and the bond of its two main spokesmen. With equal parts of compassion, analysis, and sometimes strident outrage, Frerichs and Piarroux present timelines, maps, and reports illuminating the truth. Yes, it really was “a large septic plume” emanating from the Nepalese camp near Mirebalais, they show us time and again, that first sullied the Artibonite and later, due to Haiti's woeful lack of sanitation, engulfed the entire country—“not,” as others at the time proposed, an influx of free-living *Vibrios* from brackish waters near the port of Saint Marc. By the end of the book, we are also convinced that several key actors and pundits conveniently sidestepped this truth. For readers who long to see the underside of a fast-moving epidemic including sins of omission and realpolitik, *Deadly River* does not disappoint.

Which is not to say it is perfect. For one thing, it contains a lot more “woulda, coulda, shoulda” than empathy for health workers coping with chaos. Some of the good guys are members of the Brigada Médica Cubana who rehydrated thousands at the height of the crisis; the Centers for Disease Control and Prevention (CDC), World Health Organization (WHO), and respected nongovernmental organizations (NGOs) receive far fewer plaudits. Also missing are voices of everyday Haitians.

Looking back with 20/20 hindsight, of course it would have been smart to screen Nepalese troops for cholera before they ever set foot in Haiti, just as it would have been prudent to better control their compound and sewage. “The UN meant well but boy did they screw up and has the world learned its lesson ….” is one commonly held sentiment *Deadly River* never quite concedes.

That said, no scientific history can speak for every opinion or stakeholder. In its quest for justice for Haiti, *Deadly River* serves a virtuous cause. In its staunch pursuit of truth, it also feels heroic. And talk about timing! Now that the United Nations has finally said it will strengthen its anti-cholera efforts in Haiti and compensate victims, that Portugal's Antonio Guterres has succeeded UN Secretary General Ban Ki-moon, and that a new natural disaster has seized our attention, what better moment to reexamine the past and push for redress?

For further perspective on cholera in Haiti, the *American Journal of Tropical Medicine and Hygiene* (*AJTMH*) turned to Louise Ivers, senior health and policy advisor for Partners in Health (PIH). Following the 2010 earthquake, Ivers oversaw PIH's on-the-ground cholera operations and also partnered with GHESKIO (a Haitian NGO) in pioneering studies of oral cholera vaccine, a new, cost-effective tool that is rapidly gaining adherents. Ivers now divides her time between Haiti and Boston, where she is a faculty researcher in the Division of Global Equity at Harvard Medical School and a practicing infectious diseases clinician at Brigham and Women's Hospital.

## Five Questions for Louise Ivers

**Please share some of your earliest memories of the 2010 cholera outbreak in Haiti. Where were you? What were you doing? And how did cholera affect PIH?** The day the outbreak exploded was very memorable. Early that morning, we had a meeting in Mirebalais, which included our CEO and cofounder. All of our Haitian leaders were present except for one colleague. When I texted to ask why he was late, he said he had been up all night dealing with 400 cases of diarrhea. Exhausted, he finally arrived and gave us a harrowing description of what was going on.

After that, everything moved quickly. Our hospital staff alerted the authorities; a few days later, our teams were seeing patients with diarrhea in all 12 of our clinics. The communities were afraid—hundreds of people were dying—everywhere I went there were sick people and funerals. One afternoon, we went in an ambulance to pick up one patient and returned with five more we collected along the way. Staff were exhausted, but they were absolute champions. We also partnered with other organizations. By November, there were cases in the displaced persons camps we were supporting in Port-au-Prince, including one camp with 45,000 people where we were the lead agency.

**When did everyday Haitians know or suspect that cholera had been imported by UN peacekeepers, and what happened next?** People in Haiti quickly started reporting that the UN was responsible for the outbreak. Even before the news broke in international newspapers, there were protests in Mirebalais. At the peacekeepers' base, people were carrying placards and shouting slogans against the UN. As for me … you know, in the first days and weeks we were just so busy trying to take care of thousands of people in our clinics, setting up treatment facilities, distributing water products, getting community health workers trained, lining up staff, etc., I did not pay a lot of attention to the protests except to instruct that we not stage a big shipment of buckets at the UN base as we had planned; I did not want people to think that our activities and the UN's activities were related. We already had our hands full trying to uphold peoples' trust in our management of the disease.

During that time and continuing to the present, PIH worked with the community. We held focus groups, met with local leaders, and participated in many meetings and discussions. For me, this is the real benefit of being fluent in Haitian Creole. The stories I heard were humbling. There was so much suffering and so much fear. People lost whole families to cholera in a wave of illness that they (and we) had never before experienced. *Hougans* (local Voudou priests) joined our discussions—we like to engage them in our work so they refer certain patients to the hospital. In large part, the *hougans* I spoke with quickly realized that cholera was a “maladi dokte”—an illness for doctors to treat—and referred many patients to the clinics.

**Please discuss some major lessons you learned from your work with oral cholera vaccine.** The biggest lesson I learned is that it is absolutely necessary to challenge the status quo in public health. If something does not make sense you have to ask why. In 2010, many experts were saying that OCV was too expensive, that it would not work in Haitians, that they would not come back for their second dose, that it would distract from WASH activities, and that “they” would stop washing their hands. Bottom line: these attitudes were very dogmatic and anti-poor. It was so hard to convince the powers-that-be that we only wanted to “add” vaccination to the toolkit. In high-level meetings, we were shamed when people called our solution “gold plated” or “too expensive” or “unrealistic,” but we and GHESKIO persisted in our advocacy.

Then, a year after the outbreak started, Haiti's Minister of Health transitioned; the new Minister immediately called us and said she wanted to move forward with the idea. In December, she called a meeting of partners to formally request a pilot campaign. The night before—it was a big risk as it cost us almost half a million dollars—PIH found its own funding and purchased all available doses. We were not permitted to use CDC funds—WHO created obstacles—some unknown person even called a local radio station and said we were experimenting on Haitians (since the vaccine was already WHO approved and had previously tested safe and effective in other populations, it was not true, of course). Finally, after we halted our campaign for a number of weeks and testified at a national bioethics committee, we were able to proceed. The American Red Cross gave us 1 million U.S. dollars for the vaccination campaign, a very tangible and forward-thinking investment for Haiti.

In sum, my view is neither pro- nor anti-vaccine, but it is “pro-poor,” and “pro-evidence” and “pro-using-all-the-tools-you-have.” If we had a cholera epidemic in Boston (which would never happen, but let's just say ….), we would use all available tools to stop it. But when certain people say that something is too hard or too expensive or not worth it, they tend to be people who are not truly affected. No one discussing oral cholera vaccine in the Minister's office was ever at risk of dying of cholera. But to save their families, the people who *were* at risk of dying were eager for any possible solution; that is no exaggeration.

Now the tone has changed a lot. Not only is oral cholera vaccine recommended by WHO, many countries are using it in outbreaks.

**Going forward, what are the implications of the United Nation accepting responsibility and paying reparations for a disaster caused by their own negligence; is this a good precedent or not?** I think it was critical that the UN accept responsibility. The UN caused a disaster in Haiti and their mandate is to help. I do not blame an unfortunate single soldier, but the military base should have been constructed [in such a way] to ensure a safe environment; human waste should not have leaked or been dumped into the river. From a legal standpoint, Haitians deserve justice. However, since I am not a lawyer myself, I have always focused on the moral argument, namely, that the UN should help to fix the problem they started. At times, their annual budget for Haiti has been as high as 650 million U.S. dollars, although it is less now. So it is not that the UN cannot afford to address the problem; it is a case of member states agreeing that Haiti is a priority. Not only have lives been lost, the epidemic has placed a huge economic burden on families and the health system. Haitians should also be compensated for that as well as for their suffering.

**What do you and PIH see as a realistic cholera elimination strategy in Haiti and the respective roles that civil society versus government might play in achieving that goal?** In Haiti, the central government does not have the resources to do everything that is needed and often changes, but local and regional technical managers and directors are more stable. And civil society is extremely strong. If PIH accomplishes anything it is only by staying engaged with civil society and the communities we serve. We believe that the combined, integrated approach to cholera control—in other words, combining household water and sanitation interventions with oral vaccination as an urgent means of stopping transmission—could prove successful in the next 2–5 years. In the long term, say 10 or 20 or 30 years, achieving universal access to water and sanitation would eliminate cholera altogether.

Addendum: At the time of e-publication, AJTMH learned that a million doses of oral cholera vaccine are now in Haiti for use in a new, innovative, single-dose campaign.

